# Development of a Digital Behavioral Intervention to Reduce the Consumption of Sugar-Sweetened Beverages Among Rural Appalachian Adults: Multiphased, Human-Centered Design Approach

**DOI:** 10.2196/41262

**Published:** 2023-02-01

**Authors:** Jamie Zoellner, Annie Reid, Kathleen Porter, Christina Frederick, Michelle Hilgart, Lee Ritterband

**Affiliations:** 1 Department of Public Health Sciences School of Medicine University of Virginia Christainsburg, VA United States; 2 Department of Psychiatry and Neurobehavioral Sciences School of Medicine University of Virginia Charlottesville, VA United States

**Keywords:** eHealth, human-centered design, internet-based intervention, digital technology, Model for Internet Interventions, beverages, behavioral research, rural population, mobile phone

## Abstract

**Background:**

To avoid the low engagement and limited efficacy of digital behavioral health interventions, robust human-centered design (HCD) processes are needed.

**Objective:**

The primary objective of this study was to describe a flexible, step-by-step HCD process to develop digital behavioral health interventions by illustrating *i*SIPsmarter as an example. *i*SIPsmarter is a digital intervention for reducing the consumption of sugar-sweetened beverages (SSBs) that comprises 6 internet-based cores metered out over time to deliver the program content, an integrated SMS text message strategy to engage users in reporting SSB behaviors, and an electronic cellular-enabled scale for in-home weighing. The secondary objective is to illustrate the key components and characteristics of *i*SIPsmarter that resulted from the HCD process.

**Methods:**

The methods were guided by the *Model for Internet Interventions* and by best practices in HCD and instructional design processes (eg, rapid prototype development and think-aloud protocol). The 3-phased (ie, contextual, prototype testing, end user testing phases) process followed in this study included a series of 13 semistructured one-on-one interviews with 7 advisory team participants from the targeted Appalachian user group. The interviews were content coded by 2 researchers and then deductively coded to the suggested areas of digital behavioral health interventions.

**Results:**

The participants provided rich perspectives pertaining to *i*SIPsmarter’s appearance, behavioral prescriptions, burdens, content, delivery, message, participation, and assessment. These inputs included requests for built-in flexibility to account for varying internet and SMS text message accessibility among users; ideas to resolve the issues and problems encountered when using the prototypes, including those related to navigation and comprehension of content; ideas to enhance personalized feedback to support motivation and goal setting for SSB consumption and weight; and feedback to refine the development of realistic and relatable vignettes. The participants were able to interact with multiple prototype drafts, allowing researchers to capture and incorporate feedback related to the *i*SIPsmarter dashboard, daily SSB and weight diaries, action planning, core content, interactions, and vignettes.

**Conclusions:**

Using scientific models and established processes is critical for building robust and efficacious interventions. By applying an existing model and HCD and instructional design processes, we were able to identify assumptions and address the key areas of the *i*SIPsmarter intervention that were hypothesized to support users’ engagement and promote behavior change. As evidenced by the rich feedback received from the advisory team members and the resulting *i*SIPsmarter product, the HCD methodology was instrumental in the development process. Although the final *i*SIPsmarter content is specific to improving SSB consumption behaviors among adults in rural areas, the intent is that this HCD process will have wide applications in the development of digital behavioral health interventions across multiple geographic and behavioral contexts.

## Introduction

### Background

The availability of digital behavioral health interventions has surged in recent years. However, when deployed in research trials and disseminated in real-world practice, the uptake of and engagement with many digital interventions are often lower than desired [1-3]. In turn, this results in the unrealized potential of both immediate and sustained health outcome improvements among the intended users [3]. Although numerous factors may contribute to low uptake, one of the most important is a suboptimal fit between the characteristics of the technology and the needs, skills, and context of the user. To adequately address these complex interrelationships, the development of digital behavioral health interventions should include a theory-driven, iterative, and human-centered design (HCD) process.

Importantly, there are a number of models and frameworks available to guide the digital intervention development processes [4-9]. The *Model for Internet Interventions* [4] is a key example that has been used as a basis for the development of many digital health programs [10-13]. This model can help researchers distinguish and operationalize various components of digital behavioral health interventions and identify the relationships among the components. More specifically, this model posits that to explain behavior change across digital behavioral health interventions, it is necessary to consider design-related components, areas, and elements, including user characteristics, environment, intervention content, level of intervention support, and targeted outcomes [4]. Furthermore, the *Model for Internet Interventions* highlights 8 main areas that comprise the digital health application used to deliver the intervention (ie, appearance, behavioral prescriptions, burdens, content, delivery, message, participation, and assessment). To improve the likelihood of digital health applications meeting the needs and requirements of users, HCD and instructional design processes should be applied when considering and manipulating these 8 areas.

Although this and other models and frameworks provide helpful principles and guidelines [4-9], they are not intended to be a step-by-step prescription for digital intervention development. A scoping review of 160 papers regarding research activities for participatory eHealth development processes identified a variety of methods and products [14]. However, there was no evidence of an optimal single-step approach for developing digital behavioral health intervention applications [14]. The findings from this narrative review highlighted the importance of researchers and developers selecting the most appropriate objectives for and methods for developing the context and user characteristics of their digital behavioral health interventions.

Despite the flexibility in methodological processes, the application of HCD and instructional design processes in the development of digital behavioral health interventions is consistently recommended [15]. The International Organization for Standardization defines HCD as “an approach to systems designs and development that aims to make interactive systems more usable by focusing on the use of the system and applying human factors and usability knowledge and techniques” [16]. Interdependent design activities include understanding and specifying the context of use, specifying the user requirements, producing design solutions, and evaluating the design. In addition, the International Organization for Standardization posits that six requirements must be met for an HCD process: (1) the design is based upon an explicit understanding of users, tasks, and environments; (2) users are involved throughout design and development; (3) the design is driven and refined by user-centered evaluation; (4) the process is iterative; (5) the design addresses the entire user experience; and (6) the design team includes multidisciplinary skills and perspectives [16].

Complementary to HCD concepts, instructional design processes involve designers executing cycles of continuous formative evaluation to ensure that the intervention meets the users’ needs, prior knowledge, and experience [15]. These instructional design processes should involve setting measurable learning objectives or performance requirements, assessing the users’ achievement of the targeted outcomes, and revising the program components until the desired outcomes are achieved [15,17-20]. Ultimately, the ability of digital behavioral health interventions to achieve sustained engagement and desired behavioral outcomes is enhanced when using scientific frameworks along with proven and context-specific HCD and instructional design approaches during the development process.

### Appalachian Digital Environment and Sugar-Sweetened Beverage Context

Although HCD processes are important across different contexts, the need for them is magnified in rural regions, including Appalachia. Historically, extending digital interventions into the Appalachian region has been hindered by digital divide [21,22]. However, similar to other rural American communities, Appalachia is making great strides in narrowing the digital divide and closing the rural-urban gap in home broadband internet connection and smartphone ownership [23-26]. However, little is known about how rural Appalachian adults engage with digital behavioral health interventions. As such, human-centered and instructional design processes are especially important for interventions targeting this and similarly underserved regions where access to digital behavioral health interventions has been limited.

In addition to the geographic and digital divide context of Appalachia, the behavioral context of sugar-sweetened beverage (SSB; eg, soda or pop, sweet tea, sports and energy drinks, and fruit drinks) consumption is noteworthy. SSBs are the single largest source of calories in the US diet and account for approximately 7% of the total energy intake of the US adults [27]. In Appalachia, SSB intake is disproportionately higher and accounts for an average of 14% of the total energy intake—twice as high as national estimates [28]. Consistent with both national and Appalachia-specific data showing that SSB is the largest contributor to added sugar intake [27-29], excessive SSB consumption is undeniably pervasive in Appalachia. However, there are only a few known SSB consumption–specific behavioral interventions that have used digital technologies or applied HCD processes [30-33].

### Objectives

The primary objective of this study was to describe a flexible, step-by-step approach to and an HCD process for developing digital behavioral health interventions by illustrating *i*SIPsmarter as an example. *i*SIPsmarter is a technology-based behavior and health literacy intervention aimed at improving SSB consumption behaviors among Appalachian adults. The secondary objective is to illustrate the key components and characteristics of *i*SIPsmarter that resulted from the HCD process. Although *i*SIPsmarter’s content is specific to improving SSB consumption behaviors among Appalachian adults, the intent is that the HCD process will have wide applications in the development of digital behavioral health interventions across multiple geographic and behavioral contexts.

## Methods

### Overview

**Figure 1:**
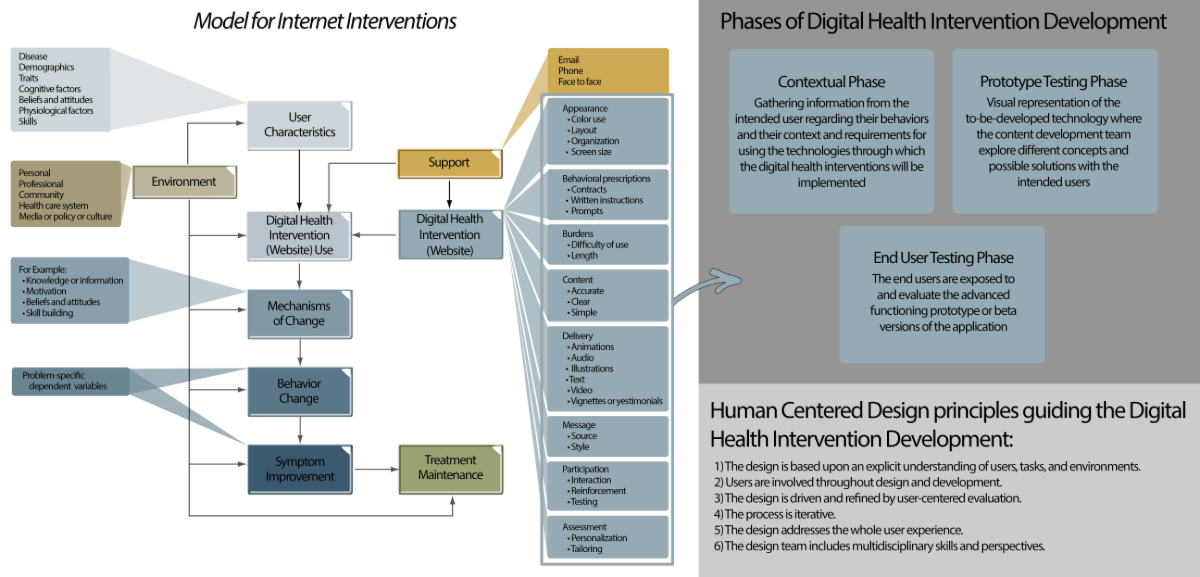
Conceptual overview and integration of the Model for Internet Interventions and best practices in human-centered design processes.

The development process for the digital behavioral health intervention *i*SIPsmarter was guided by the *Model for Internet Interventions* [4] and by best practices in HCD [16] and instructional design processes [17-20], which is being evaluated in a randomized controlled trial (Trial Registration: NCT05030753). Figure 1 illustrates the conceptual integration of this model and these processes, along with key definitions. Specifically, the development process of *i*SIPsmarter included 13 semistructured interviews across three nonsequential iterative phases: (1) contextual, (2) prototype testing, and (3) end user testing phases.

This formative, flexible, and step-by-step approach allowed the advisory team participants to provide insights at all phases of the development process and interact with multiple prototype drafts developed by the content development team (CDT).

The following sections describe the considerations for the CDT, advisory team participants, adaptation context, *i*SIPsmarter intervention, and adaptation process. Subsequently, the 3 phases of data collection and the data analysis strategy are described.

### Ethical Considerations

This program evaluation project involved activities that do not represent human participant research and, therefore, did not require submission to the institutional review board (Human Research Protection Program Standard Operating Procedures [34]).

### The Role of CDT

The development of digital behavioral health interventions should be conducted by a multidisciplinary CDT [15,16,35]. This includes subject matter experts, behavioral psychologists, web designers, instructional designers, and developers and programmers.

The multidisciplinary CDT involved in the development of *i*SIPsmarter included 4 doctoral-level researchers: 2 (50%) SSB content experts who had led prior SSB behavioral intervention trials in the targeted Appalachian region, 1 (25%) expert on digital behavioral health technology interventions, and 1 (25%) instructional design expert. This team was further supported by research staff experienced in nutrition, public health, and digital health development and implementation, including those with expertise in the design and creation of robust user interface and user experience. For the duration of this HCD process, the team met weekly to collaboratively draft advisory team interview guides with clear objectives, develop prototypes, and respond to feedback from the participants. Likewise, the CDT also wrote content for the *i*SIPsmarter cores, developed media-rich interactions and videos, drafted personas and vignettes, and developed an integrated internet-based platform.

### Advisory Team Participants

When developing digital behavioral health interventions, the selection of advisory team members is an important decision. Ideally, the participants should represent the intended end user of the intervention in terms of demographic and cultural characteristics, health literacy and digital literacy skills, geographic location, and patterns of the targeted behaviors [16].

The advisory team members involved in the development and design of *i*SIPsmarter were a convenience sample of prior SIP*smart*ER participants who resided in the Appalachia region. The advisory team members also had to have internet access and the ability to review the *i*SIPsmarter program materials on the web. These members were diverse in terms of age (21 to 60 years), gender, and socioeconomic status. Of the 15 prior SIP*smart*ER participants invited, 7 (47%) joined the advisory team. These participants were involved throughout the intervention design and development processes, with the first interview conducted in February 2019 and the final interview in December 2021. In total, the advisory team members were compensated with up to US $200 in electronic gift cards for their participation.

### Adaptation Context

*i*SIPsmarter is a technology-based behavior and health literacy intervention aimed at improving SSB behaviors among Appalachia adults [36]. *i*SIPsmarter was adapted from the evidence-based intervention SIP*smart*ER [37]. SIP*smart*ER is a 6-month intervention that includes 3 in-person small-group classes, 1 live teach-back call, and 11 interactive voice response (IVR) calls; the intervention also comprises a 12-month maintenance phase that includes monthly IVR calls (refer to the database Evidence-Based Cancer Control Programs for details [38]). The classes cover key behavioral content and action planning, whereas the IVR calls engage users in reporting SSB intake and personal action planning. Several formative research projects among Appalachian adults initially guided the development of SIP*smart*ER [39-41]. The content and strategies of SIP*smart*ER are guided by the theory of planned behavior and health literacy, numeracy, and media literacy concepts [42-46]. In brief, SIP*smart*ER was found to be effective at reducing and maintaining SSB consumption behaviors through a full-scale randomized controlled trial and a pilot dissemination and implementation trial conducted in collaboration with the Virginia Department of Health districts in Appalachia, Virginia [29,37,47-58]. Although SIP*smart*ER had been highly successful in reducing SSB consumption, transitioning from its original structure to a fully digital modality presented an opportunity to focus on optimizing scalability and reach.

At the onset of developing *i*SIPsmarter, the CDT had ample experience understanding and intervening on SSB consumption in the targeted region and had over a decade’s worth of rich qualitative, observational, and experimental data from the previous SIP*smart*ER trials. As such, content and behavior change techniques related to SSB consumption were relatively well established when embarking on the digital intervention development. However, little was known about the opportunities, barriers, and access to technology in the targeted population for intervention delivery or data collection purposes. Similarly, weight self-monitoring and the use of cellular-enabled scales to encourage in-home weighing had not been previously explored in the targeted population and region. Therefore, the intention of the *i*SIPsmarter adaptation process was largely focused on changing the mode of delivery and adding key content around weight while maintaining other core components and the cultural relevance for the intended Appalachia target audience.

### *i*SIPsmarter Description

In its final form, *i*SIPsmarter comprises 6 internet-delivered interactive cores, which are metered out sequentially over time, with a new core becoming available 1 week after completion of the previous core; an integrated SMS text message strategy to engage users in tracking and reporting SSB consumption behaviors; and an electronic cellular-enabled scale for in-home weighing [36]. At the end of cores 2 to 6, the users evaluate their SSB and weight diary data and set a personalized SSB consumption and weight action plan for the upcoming week. After the sixth core is completed, there is a recurring maintenance core where users can continue behavioral tracking and personalized action planning. To assist with mastering key content and behavioral strategies, the cores also include PDF resources that users can view or print. *i*SIPsmarter is highly interactive and contains a media-rich format of text, audio, graphics, animation, interaction, and video. Vignettes are woven throughout the intervention, which include stories (based on the experiences of past SIP*smart*ER participants) to model and describe situations related to setting goals, behavior changes, resolving barriers, and encountering slips. Finally, *i*SIPsmarter includes a stepped care engagement strategy (ie, human-supported text messages, followed by phone calls if needed) to provide encouragement, technical assistance, and strategies to promote core completion. The adaptation processes, HCD approach, and findings leading to the final content and structure of *i*SIPsmarter are further detailed in the subsequent sections.

### Areas of Adaptation and the Digital Health Intervention Development Process

The *Model for Internet Interventions* highlights 8 areas to consider when developing digital behavioral health interventions (Figure 1) [4]. These areas are defined and illustrated within the adaptation context for *i*SIPsmarter.

#### Content and Messages

Content refers to the actual intervention information and may be the single most important component of the program [4]. Focus is placed on the style and source of messages, which are theorized to impact user engagement and other mechanisms of change, including knowledge acquisition and motivation. [4]. Similar to SIP*smart*ER, *i*SIPsmarter is grounded in the Theory of Planned Behavior and health literacy, numeracy, and media literacy concepts. In addition, the scientifically grounded core content, key learning objectives, and behavior change techniques of the digital *i*SIPsmarter intervention are similar to those of the original evidence-based SIP*smart*ER intervention [36,37]. The main difference between the two is the addition of evidence-based weight self-monitoring and weight-related strategies to *i*SIPsmarter. Therefore, the development process focused heavily on weight-related content and messages. In addition, all *i*SIPsmarter content was written using clear communication strategies and with the goal of achieving <eighth grade reading level. Specifically, all user-facing core content pages were entered into the Readable website to determine the readability score using the validated Flesch-Kincaid Grade Level formula, which considers sentence length and word length [59]. *i*SIPsmarter passages were revised in up to 2 rounds to improve readability (ie, reduce sentence length and reduce multisyllabic words). This process resulted in 90% of the *i*SIPsmarter core content pages meeting the criteria of <eighth grade level.

#### Delivery and Participation

Delivery refers to the form in which the intervention content is disseminated and includes text, audio, illustrations or graphics, animations, video, and vignettes or stories or testimonials [4]. Participation is focused on the program’s ability to engage and involve the user in the intervention [4]. Within each *i*SIPsmarter core, videos and interactions were developed using gamification principles to encourage the users to engage in learning and practicing key behavioral strategies and techniques [60]. The intervention content prioritized both interactions focused on skill development and personalized reinforcement to drive behavior change (refer to Table 1 for details). To replicate the role modeling and observational learning that occurred during the SIP*smart*ER small-group classes, personas and vignettes were developed for *i*SIPsmarter. Personas are defined as user archetypes that represent the characteristics of future users or actual people from a targeted group [14]. Vignettes are narrative stories that illustrate key situations and real-life scenarios faced by individuals and problem behaviors that the intervention aims to improve. The development of personas and vignettes was a multistep and iterative process. First, based on the CDT’s experience with past SIP*smart*ER participants and intended users of the digital *i*SIPsmarter intervention, 9 personas were identified and developed to represent the range of traits and SSB consumption change patterns that the CDT witnessed among SIP*smart*ER participants. These personas detailed numerous user characteristics, such as (1) demographics and family or social characteristics, (2) SSB consumption change patterns and barriers, (3) weight-related patterns and barriers, (4) motivation level and perceived behavioral control, and (5) the use of planned digital technology components. Then, journey maps were created to share with the advisory team participants. These journey maps were single-page snapshots modeling each persona’s potential path through *i*SIPsmarter, including experiences, successes, and challenges with improving SSB consumption and weight behaviors. Feedback was solicited from the advisory team participants. On the basis of the participants’ insights, the personas were narrowed down and further refined. Next, vignettes (ie, narrative stories) were developed for each persona to model and describe situations related to setting goals, behavior changes, resolving barriers, and encountering slips. Finally, vignettes were mapped to key content through each of the *i*SIPsmarter cores and several of the interactions.

**Table 1:** Final *i*SIPsmarter overview: asset summary, user objectives, interactions or video descriptions, printable PDFs, and user summaries.

Core	Overview and number of assets	User objectives	Interactions or videos description
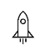 Core 1: getting ready	Core content screens: 26 Action planning screens: N/A^a^ Interactions: 4 Videos: 3 Vignettes: 16 Printable documents: 3	See how *i*SIPsmarter works and what to expect List my personal reasons for joining *i*SIPsmarter Discover what counts as a sugary drink Recognize portion sizes of sugary drinks Recall my typical sugary drink patterns Track my sugary drinks and weight for my *i*SIPsmarter Diaries	Interactive questions to raise awareness on SSB^b^ availability, SSB costs, and the amount of sugar in SBBs Interactive content to show successful results from the previous SIPsmartER intervention Sorting game to recognize and practice what counts as an SSB Sorting game to recognize the portion size of SSBs Three short videos to highlight the importance of tracking SSB consumption, the importance of tracking weight, and how to use *i*SIPsmarter to track SSB consumption and weight
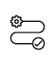 Core 2: making a plan	Core content screens: 29 Action planning screens: 22 Interactions: 4 Videos: 1 Vignettes: 12 Printable documents: 8	View the recommendations for sugary drinks Recognize the health risks of too many sugary drinks Explore red-light, yellow-light, and green-light drink categories See the health benefits of non-sugary drinks Evaluate my weight and see a healthy weight range Set a personal Action Plan to help meet my sugary drink and weight goals	Interactivity to rate the importance of and confidence in decreasing SSB consumption, with personalized feedback Interactive game to realize the amount of sugar in SSBs and equivalent sugar packets per day Interactive body map to recognize the health risks and key health facts associated with the consumption of too many SSBs Video to learn about how the consumption of too many SSBs impacts the body and leads to health risks over time Interactivity to illustrate the connection between the consumption of SSBs and weight over time
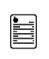 Core 3: using numbers	Core content screens: 23 Action planning screens: 8-12 Interactions: 1 Videos: 1 Vignettes: 9 Printable PDFs: 3	Recognize my calorie and energy balance needs Identify my limits on added sugars Apply information from food labels to identify sugary drinks Set a personal Action Plan to help meet my sugary drink and weight goals	Video to highlight the key components and application of the nutrition facts label, which includes grams of added sugars, servings per container, serving size, and ingredient list Four-part interactivity to apply one's skills in reading nutrition labels, identifying different names for sugars, and sorting drinks using the nutrition facts label and to learn tips that can be applied when a drink does not have a nutrition facts label
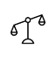 Core 4: balancing choices	Core content screens: 33 Action planning screens: 8-12 Interactions: 1 Videos: 0 Vignettes: 9 Printable documents: 6	Explore red-light, yellow-light and green-light food categories See how red-light drinks and foods can create imbalance in my diet Recognize how to plan ahead to balance my choices Practice reducing my red-light drink and food choices Discover the health benefits of physical activity Set a personal Action Plan to help meet my sugary drink and weight goals	Two-part interactivity to identify red-light foods that are consumed often and select red-light foods that can be removed, replaced, or reduced
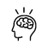 Core 5: thinking critically	Core content screens: 26 Action planning screens: 8-12 Interactions: 2 Videos: 0 Vignettes: 10 Printable documents: 2	Recognize my sugary drink choices may be swayed by marketing Identify common marketing tactics used to sell sugary drinks Discover the goal of sugary drink companies is to make me spend money Evaluate the costs of sugary drinks Analyze, critique, and modify sugary drink ads Set a personal Action Plan to help meet my sugary drink and weight goals	Interactivity to recognize that, in the context of SSB marketing, people’s attention is the product SSB commercial with interactive questions and feedback to help the participants see through marketing and think critically about advertisements
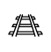 Core 6: staying on track	Core content screens: 23 Action planning screens: 8-12 Interactions: 1 Videos: 0 Vignettes: 12 Printable documents: 2	Evaluate my sugary drink and weight program goals Learn how to handle slips in my red-light sugary drink and food choices Focus on strategies to prevent prolonged relapses Check my understanding of key *i*SIPsmarter concepts Set a personal Action Plan to help me stay on track with my changes	A 25-item interactive quiz to help the participants review and check their understanding of the key *i*SIPsmarter concepts in cores 2 to 6

^a^N/A: not applicable.

^b^SSB: sugar-sweetened beverage.

#### Behavioral Prescriptions and Assessments

Behavioral prescriptions instruct the user on how to address the targeted problem. They are designed to increase commitment and boost adherence and may include, for example, behavioral contracts as well as automated and personalized prompts (eg, emails and SMS text messages) [4]. Assessment refers to the ability to measure the needs of the user, personalize the program, and provide tailored content and recommendations [4]. In the case of *i*SIPsmarter, the personalized action planning process, behavioral monitoring, and personalized feedback loop were transitioned from the original format of small-group classes and IVR calls in SIP*smart*ER to a fully digital format. The web development team built *i*SIPsmarter on a proprietary software platform called the Research Infrastructure Containing eHealth (RICE) interventions, developed for building digital health programs. The RICE platform integrates all aspects of a digital intervention for a seamless user experience and research administration. *i*SIPsmarter provides an opportunity to extend the technological capability of the intervention infrastructure by incorporating SMS text message and sensor integration, specifically, the BodyTrace scale (BodyTrace, Inc). Daily SMS text message prompts are sent to encourage users to report the of ounces of SSBs consumed the previous day. In addition, an electronic cellular-enabled BodyTrace scale is provided for weight data collection. Users are encouraged to step on the scale daily, with a minimum threshold of 3 days per week, to receive personalized feedback. The integration of the internet-based platform, SMS text messages, and cellular-enabled scales created an integrated experience, whereby users can log into the internet-based platform and view their progress through the cores, along with synced personal diary data (SSB consumption and weight) on their *i*SIPsmarter dashboard. When appropriate, gamification principles are also applied when providing tailored feedback and recommendations (eg, cues when cores are complete and encouraging feedback when SSB and weight diaries are entered) [60].

#### Burdens

Burdens are specific to the intervention content and can include problems related to use, such as poor application navigation and program length [4]. In *i*SIPsmarter, understanding the burden of the users was prioritized when developing features associated with diary tracking, dashboard navigation, the action plan process. Given that goal setting, planning, self-monitoring, and feedback are among the most important behavior change techniques, understanding and resolving potential user burdens with the digital content was especially important. In addition, attention was paid to the overall length of each core and to balancing the amount of content with the projected user fatigue.

#### Appearance

Appearance refers to the look and feel of the application and can include the use of color, page or screen layout, organization of content, and screen size [4]. For *i*SIPsmarter, a graphic designer developed key images and icons with a consistent theme and style. These graphics were applied throughout the intervention, including all core screens and printable documents.

### Additional Components of the Digital Health Intervention Development Process

The *Model for Internet Interventions* (Figure 1) also illustrates other design-related components and elements (eg, *support, user characteristics, environment,* and *digital health intervention use*) [4]. Many of these components were considered in the *i*SIPsmarter development process. As an example, the model describes *supports* (eg, email, phone, and face-to-face) that can directly impact users’ adherence to digital health applications. During the development and design of *i*SIPsmarter, interviews were conducted with the advisory team members to assess their understanding and perceptions of program reminders and gather inputs on the timing and content of stepped care messages.

### Data Collection

#### Overview

The *i*SIPsmarter components were developed using a flexible and iterative 3-phased HCD process. This process included semistructured interviews with the advisory team participants. For each interview, the CDT collaboratively developed key objectives and designed interview guides during weekly team development meetings. One of the researchers drafted the initial questions based on the focus area and agreed upon objectives. Subsequently, other team members provided feedback until a final version was agreed upon. All the interview guides used open-ended questions and probes. The interview content was prioritized by the current development activities. Therefore, it was possible to follow an ongoing, iterative, HCD process that matched the steps and pace of the program development. One or 2 researchers led the audio-recorded interviews. The interviews were completed over telephone and via Zoom (Zoom Video Communications Inc). The sessions ranged from 45 to 60 minutes.

#### Contextual Phase

The contextual phase of the HCD process involves gathering information from the intended users regarding their behaviors and their context and requirements for using the technologies through which the digital health interventions will be implemented [5,16,18]. Given the CDT’s ample experience intervening in SSB consumption among frequent consumers in the targeted region and a deep understanding of many of the components of the *Model for Internet Interventions* (ie, user characteristics, mechanisms of change, and behavior change) [4], the 4 interviews comprising the contextual phase largely focused on important technology-related factors associated with potential *i*SIPsmarter use as well as research evaluation components.

The initial advisory team meeting explored participants’ technology ownership and use, internet availability and use, and perceived benefits of and barriers to receiving a digital behavioral health intervention. The 3 subsequent contextual interviews assessed the participants’ understanding and perception of program reminders and gathered feedback on the timing and content of stepped care messages. Likewise, the participants’ experiences with daily weighing on scales and perceptions toward using cellular-enabled scales for a digital health intervention were evaluated. Finally, the participants’ feedback on the research outcome data components that were assessed was solicited via an internet-based survey, telephone calls, and cellular-enabled scales, all of which were completed by the participants in the comfort of their homes.

#### Prototype Testing Phase

Prototype testing includes the visual representation of the to-be-developed technology, where the CDT explored different concepts and possible solutions with the intended users. Testable prototypes can take many forms, such as paper prototypes, mock-ups, and wireframes (eg, a skeletal framework of an interface, usually a website or other applications) [5,16,18]. Prototype testing is a critical phase for iteratively addressing and responding to user feedback across the suggested areas of digital behavioral health interventions (ie, appearance, behavioral prescriptions, burdens, content, delivery, message, participation, and assessment) [4].

The *i*SIPsmarter prototypes developed in this phase were paper- and web-based sketches that illustrated planned scenarios. The participants were sent links to the web-based prototypes, which included preprogrammed pages that displayed various feedback points as the participants moved through the content. This intentional rapid prototyping allowed the participants to interact with multiple prototype drafts and allowed the researchers to incorporate iterative feedback from the participants. The think-aloud method, which is a common approach to assessing the usability of digital health interventions and involves the participants verbally narrating their thoughts when completing a task related to the prototypes, was used. The researchers guided the process and asked the participants to complete specific tasks. Open-ended questions allowed for a robust understanding of usability and functionality. The interviewers documented where the participants encountered problems and difficulties using the prototypes.

This phase included 6 interviews to assess the participants’ comprehension and experience and the overall prototype functionality. The prototypes largely focused on the design and content of the *i*SIPsmarter user dashboard, daily SSB diary, action planning, and vignettes. The prototypes of the *i*SIPsmarter user dashboard and daily SSB diary were shown to the participants multiple times. The participants were asked to interact with the dashboard prototypes to collect data on their comprehension of the dashboard interface. This included navigating the dashboard to start a core (lesson) and add a daily SSB diary. Diary prototypes were also tested to collect data on the users’ understanding of adding daily SSB ounces. In addition, the participants were presented with SMS text message screenshots to assess their comprehension of daily SSB consumption tracking features. Similarly, prototypes of SSB and weight action plans were presented to the users to inform the development of a key intervention behavior change technique, personalized goal setting. Moreover, as previously mentioned, the journey maps were shared with the advisory team participants, and feedback was solicited to help narrow down, refine, and develop the vignettes associated with the personas.

#### End User Testing Phase

In this phase, the advanced functioning prototype or beta versions of the application are exposed to the end users and evaluated [16,18]. This phase can be carried out in a controlled laboratory setting, yet it is typically more useful when the intervention is field tested in the user’s own environment.

For *i*SIPsmarter, once the prototypes transitioned into the web-based RICE platform, the program-enabled website underwent numerous rounds of internal review by the CDT for clarity of content, flow, and transitions and for addressing programmatic bugs. Subsequently, the end user testing phase began. The advisory team participants were given access to the *i*SIPsmarter website in their own environment. They completed the cores, including the embedded interactions, videos, and vignettes. Furthermore, they tracked their SSB intake and weight using the SMS text message feature and electronic cellular-enabled scale, respectively. A total of 3 semistructured interviews explored the usability and functionality of and user experience and satisfaction with the *i*SIPsmarter website.

### Data Analysis

After each round of interviews, the researchers who conducted the interviews reviewed the audio transcripts and created interview summary documents that summarized each participant’s response to each question. Following each interview series, these summary documents with screenshots were shared with the CDT to incorporate feedback into the ongoing intervention content and programmatic development.

The analysis and data interpretation process involved several steps [61,62]. First, the 2 researchers who conducted the interviews reviewed the summary documents and independently identified key takeaway statements for each of the 13 interview rounds. Then, they met to build consensus and finalize the interview-level takeaway statements. Second, 2 researchers independently examined and deductively summarized the interview-level takeaway statements as higher-level findings and incorporated them into all 3 HCD phases, as aligned with the *Model for Internet Interventions.* During this second step, they also used CDT meeting minutes and artifacts from the development process (eg, prototype versions and drafts of core content) to inform higher-level findings. Again, the 2 researchers met to build consensus on the overarching phase-level findings. Finally, to illustrate the phase-level findings, key quotes from the advisory team members were extracted from the transcripts.

## Results

Multimedia Appendix 1 illustrates the goals of the 13 semistructured advisory team interviews along with key interview-level takeaways. The phase-level findings, as aligned with the *Model for Internet Interventions*, are further summarized below.

### Contextual Phase

Interviews 1, 3, 11, and 12 focused on the contextual and technology-related aspects of intervention delivery and data collection. The following key findings emerged:

- To promote *participation*, the participants described the need for built-in flexibility to account for varying levels of internet and SMS text message accessibility (*digital health intervention use*).
- The participants reported the need for accountability and personalized *assessments*.
- Plans for stepped care contacts were viewed as a helpful and important intervention component (*support*) that could boost adherence and core completion (*behavioral prescription*).
- The participants saw value in a weight monitoring component to promote reinforcement (*participation*) and personalization (*assessment*), yet barriers to weighing varied based on *user characteristics* and *environmental* factors, such as limited cellular service to transmit weights.
- The intervention enrollment procedures were easy to understand and complete.

These findings are illustrated by several key quotes from the advisory team members. One of the members expressed that they face difficulty in responding to SMS text messages owing to a lack of signal but that they would be able to email:

...I can receive her message but...I couldn’t answer her as prompt[ly] as I should or whatever. I’d have to find a place where I could get enough signal to send her back a reply. But with my home computer, if I get on there and have an email that I need to check, I can go ahead, you know, and go through and do it.

In reference to monitoring weight on the *i*SIPsmarter dashboard, one of the members said the following:

I think it’s a good reminder...to cut back on your sugary drinks...if you’re able to see your weight up there.

### Prototype Testing Phase

**Figure 2:**
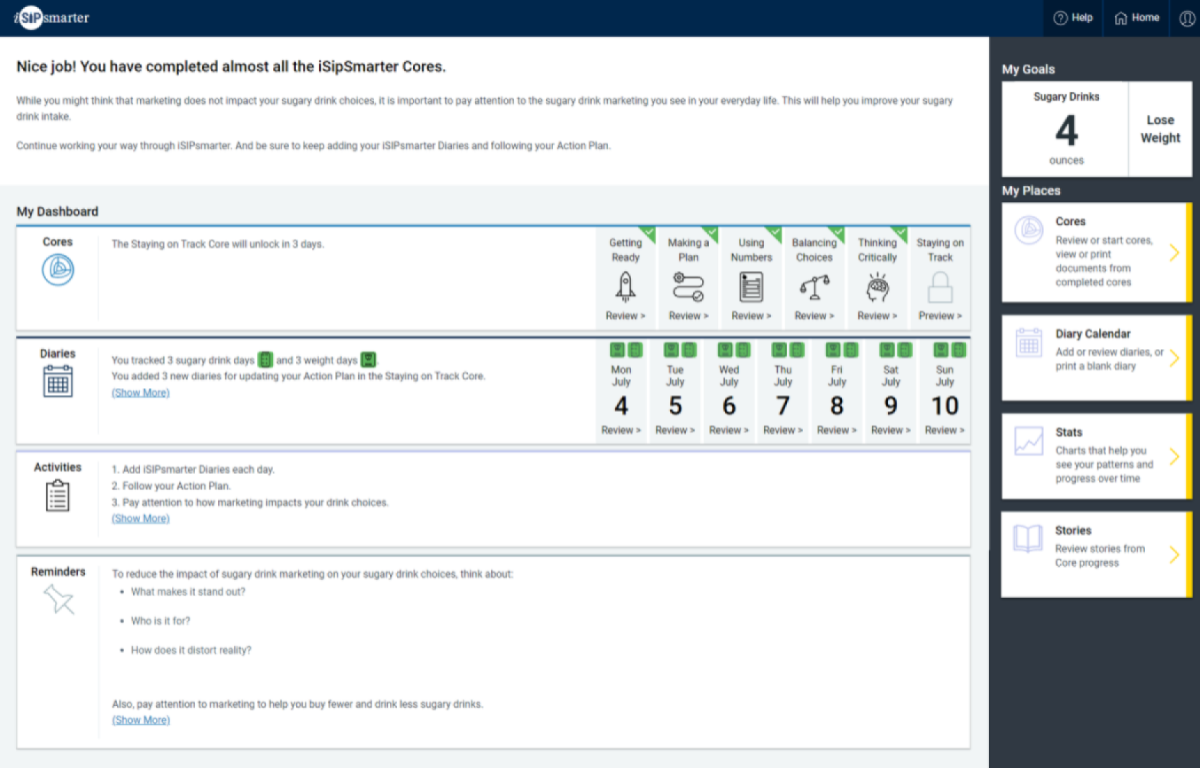
Final dashboard screenshot.

**Figure 3:**
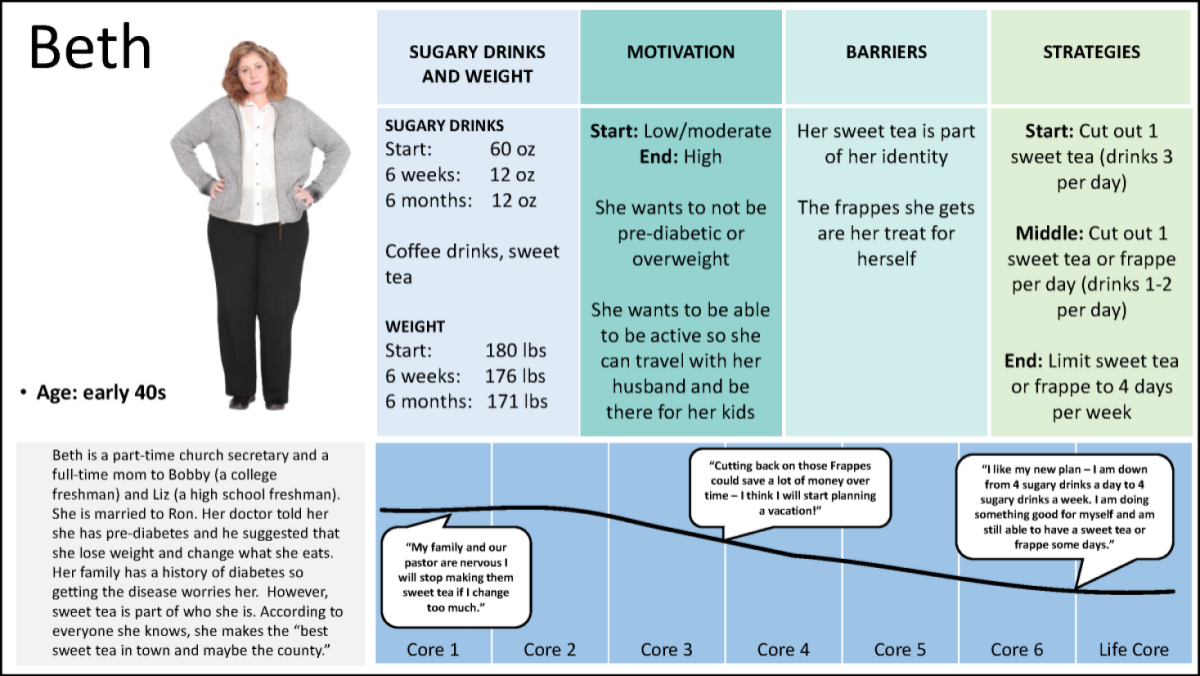
Example journey map shared with the advisory team participants.

The prototype testing phase allowed the CDT to iteratively address and respond to the user feedback. Interviews 2 and 8 focused on the dashboard and diaries; interviews 5, 6, and 7 were dedicated to the action planning concepts; and interviews 4, 5, and 6 focused on the personas. The final dashboard illustrated in Figure 2 contains a few examples of key intervention components, and a sample action plan is shown in Multimedia Appendix 2. For the persona-focused interview, 9 persona journey maps were shared with the advisory team participants, one of which is illustrated in Figure 3. On the basis of the participants’ insights into and reflections on the journey maps, the 9 initial personas were narrowed down to 6 and then further refined.

The main phase 2 findings are summarized as follows:

- The participants felt that flexibility in diary tracking methods and resources (eg, SMS text message, web-based tracking, drink cards, and paper diaries) would enable high engagement and *participation* among the users in tracking their SSB consumption and weight (*behavioral prescriptions*). For example, the participants who prefer paper diaries could first log their daily diary data via pencil and paper. Then, they could log into their dashboard weekly and back enter their diary data. Alternatively, the participants who prefer the digital methods could respond to the daily SMS text messages or email messages.
- Through multiple iterations, the dashboard became easy to navigate (low *burden*), with clear *content*, and contained helpful tailored user information (*assessment*), including visual cues to signify the completion of the core and diary tasks (*appearance*).
- The action planning process was easy to navigate (low *burden*); the *content* and *delivery* features were clear; a personalized and tailored feedback was perceived to be helpful when setting and monitoring SSB and weight goals (*behavioral prescriptions*, *assessment*, and *messages*).
- Weight-related *messages* and *content*, including barriers and strategies, were relatable and easily understood.
- The vignettes were perceived as realistic and relatable, indicating an effective *delivery* approach for conveying key *content*.

Highlighting different patterns in SSB consumption behaviors among the vignettes was identified as a helpful *messaging* approach to improve the personalization and tailoring of *content*.

The following key advisory team member quotes support the findings:

In reference to the ability to navigate the action planning process, one of the members said the following:

Everything else was pretty easy to understand. It was straightforward. It wasn’t lengthy as far as, like, a whole bunch of text that you needed to read. It provided really good examples for people to go by.

In reference to the personas being realistic and relatable, the following was said:

Kim because...She’s a single mom working full time. Busy schedule. Kind of just drink most of the day because she was too busy to eat...That was basically my story. So,...I can totally relate to that one. Wanted to be a healthy role model for her kids.

### End Users Testing Phase

Interviews 9, 10, and 13 focused on the end user testing. Table 1 highlights the final *i*SIPsmarter overview that was evaluated in this phase, including the asset summary, the user objectives, and a description of interactions or videos. The cores and interactions were developed and programmed in a manner that allows the users to go back and review the content and repeat the interactions to master skill development. Related to action planning, the underlying programmatic structure in cores 3 to 6 and the maintenance core are identical. However, the personalized feedback loops change based on the user’s diary data, progress toward their prior goals, and their new goals. The final SSB message bank includes 14 barriers and 80 strategies, and the weight-related message bank includes 13 barriers and 115 strategies. Therefore, each time a user completes an action plan, the personalized feedback has the potential to look very different (Multimedia Appendix 2).

On the basis of phase 2 findings, the CDT fully developed 6 vignettes and mapped key content through each of the *i*SIPsmarter cores and several interactions. Table 2 illustrates a final vignette, along with several examples of how the vignette is integrated across the cores.

The following phase 3 findings emerged when the participants were allowed to access the programmed intervention in their own environment:

- The overall *appearance* of *i*SIPsmarter was well received, including layout, organization, iconography, graphics, visuals, and color use.
- The participants reported high satisfaction with how the *messages* and *content* were *delivered*, including interactions, animations, videos, vignettes, and illustrations.
- Few *burdens* were reported, as the participants found the cores to be enjoyable, easy to navigate, user friendly, not overly text heavy, and of an acceptable length. Minor bugs and glitches were identified and resolved before launching the intervention.
- *Participation* was enhanced by the built-in flexibility for diary tracking.
- The *behavioral prescriptions* and personalization of *assessments* were well received, particularly the action planning process and automated emails and SMS text messages for reminding the participants to track their diaries.

A few key quotes from the advisory team member have been illustrated to support these findings. In reference to the interaction aimed at teaching and reinforcing the amount of sugar in drinks, one of the members said the following:

When I clicked on...the container that you drink out of the most...and it showed you the little packets of sugar out there. I was like, Oh my gosh, when I seen that it really hit and I thought, No way do you need that.

In reference to the personalized feedback, the following was expressed:

[I]t’s always good to visualize because, you know, it’s hard to kind of think back to what I did you know last Thursday. But on something like this where I can see, OK, I had 12 ounces as last Thursday, I can kind of pull back in memory of the exactly OK, what I did those days that either led me to only have this amount or led me to end up drinking this amount. So it...put those numbers into context a lot better than it would if you just said is average, this is your high this your low.

In reference to the overall *i*SIPsmarter user experience, the following was said:

...[T]he website itself—that’s the thing that stood out the most to me. It’s well designed. It’s easy to navigate. It’s not too complicated. Everything’s well-organized...Everything is explained well, and it’s simple and concise and to the point. I like the level of the language that’s used. There’s not a whole lot of like jargon or extensive terminology or anything like that that might shy people away from participating. I think the readability of it is really well.

**Table 2:** An example vignette summary from *i*SIPsmarter.

Name and image	Profile	Example stories throughout the cores
Beth 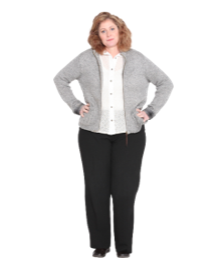	Beth is a 53-year-old married homemaker and part-time church secretary. She has two grown kids who live away from home. She cares for her elderly father Sugary drink pattern: Total ounces=64 ounces per day 16 ounces frappe, three 16 ounces glasses of sweet tea throughout the day Reasons for drinking sugary drinks: likes the taste; enjoys making sweet tea for family and friends; it’s a stress reliever Physical activity: sedentary-has a gym membership but rarely goes; wants to be more active but has a hard time fitting it all in while caring for her father Health status: just told she has diabetes and has gained 20 pounds in a year Reason for joining *i*SIPsmarter: wants to manage the diabetes so she can be healthy, travel with her husband and take care of her family Quote: “It’s just part of who I am”	Core 1—Texting Makes Tracking Easy: The daily text makes tracking my sugary drinks easy. I simply respond to the text with my total sugary drink ounces from yesterday. Also, I like seeing everything in one place when I sign in to *i*SIPsmarter. Core 2—Getting My Diabetes Under Control: I was shocked when my doctor told me I had diabetes. My doctor told me getting to a healthier weight and making better food and drink choices were important to manage my diabetes. I started thinking about my health more. I know I have to take care of myself to keep taking care of my family. I saw that I drank too many sugary drinks and learned its impact on my diabetes and weight. I knew cutting back on sugary drinks was going to be hard. Sweet tea and my frappes were just part of my routine. Core 3—Getting Help from Others to Stay Accountable: I work part time for my church, and the staff often goes out to eat together. When eating out with my coworkers, it was harder to stick to my sugary drink goals. I told my co-worker Mary about *i*SIPsmarter and my goals and asked her to help me. Getting her support, helped me stay accountable. Core 5—Could be Saving Money for College Tuition: My husband and I are helping our son with college costs so he doesn’t finish school with too many student loans. We are always looking for ways to save money. The frappes I was getting every day were $5 each day or $1,820 a year! This amount of money will definitely help cover some of his college costs.

## Discussion

### Principal Findings

This paper presents a flexible, step-by-step approach to and an HCD process for developing digital behavioral health interventions using *i*SIPsmarter as an illustrative example. By applying the *Model for Internet Interventions* [4] as well as best practices in HCD [16] and instructional design processes [17-20], we have been able to clarify assumptions and address key areas of the *i*SIPsmarter intervention that were hypothesized to support participants’ engagement and promote behavior change. As evidenced by the rich feedback received from the advisory team members, the human-centered methodology was instrumental in our development process. Likewise, the value of our robust process is exemplified by our resulting user-informed, high-quality products (eg, *i*SIPsmarter core components, vignettes, dashboard, and personalized action plan). Our approach can be interpreted within the context of the findings of recent narrative scoping review by Kip et al [14], in which a variety of methods (eg, interviews, focus groups, questionnaires, card sorting, and usability testing) and products (eg, prototypes, personas, and behavior change strategies) used for participatory eHealth development processes have been highlighted. Similar to the conclusions of Kip et al [14], our approach illustrates the importance of researchers and developers using the most appropriate methods to match their objectives and user characteristic context.

In terms of the *i*SIPsmarter development process, some of the biggest challenges faced when transitioning from the original group class structure to the digital structure include (1) replicating engagement provided by participant-to-participant and participant-to-instructor communication and relationships; (2) balancing the demands and cognitive load of the behavior change content (especially planning for the potential low health literacy skills of our targeted users) and trying to mimic visual, experiential, and hands-on class-based activities; (3) building flexibility to account for varying levels of internet and SMS text message accessibility among users; (4) creating a seamless user experience by integrating internet, SMS text messages, and sensor information; (5) operationalizing all aspects of the intervention content for digital delivery; and (6) automating all elements while ensuring that all permutations were considered. Similar challenges have been highlighted in a few other digital behavioral health intervention adaptation papers [14]. However, these issues are often overlooked and underreported in the literature, which may partly explain the low uptake of digital behavioral health interventions as well as the suboptimal fit between the characteristics of the technology and user needs. Simply converting evidence-based content traditionally delivered face-to-face to a web-based or digital format, without adequate attention to these and other challenges, is an insufficient approach to engaging the intended users or promoting improved health outcomes. Researchers and developers should rely on the established models and frameworks, such as the *Model for Internet Interventions* [4-9], to help anticipate and guide key decisions when embarking on the development of digital behavioral health interventions.

Although several behavioral interventions targeting SSB consumption reduction among adults have been developed and evaluated [30], only a few have used scalable digital approaches. Moreover, only one other known adult-focused digital intervention targeting SSB consumption reduction has applied user-centered methodologies in the formative phases of intervention development [31]. Similar to our study, the study by Tonkin et al [31] focused on disadvantaged and nonurban adults, and its findings revealed the importance of understanding the available technology and patterns of its use as well as participants’ preference for stories, role modeling, and gamification, which foster engagement with the intervention. In addition, digital weight self-monitoring has become a cornerstone of many weight-related behavioral interventions, and greater adherence to self-monitoring is associated with better outcomes [63-65]. Unfortunately, consistency and disengagement in digital weight self-monitoring are known to be problematic [66]. Although several studies have investigated experiences of self-monitoring at the conclusion of interventions [67,68], there is a dearth of published studies that have applied HCD processes in formative intervention development stages to understand and build-in behavioral strategies to address potential personal and environmental barriers to digital self-monitoring [69].

On the basis of our experiences with *i*SIPsmarter, we offer 6 broad considerations for other teams developing or adapting digital behavioral health interventions (Textbox 1).

In terms of study implications, the efficacy of *i*SIPsmarter in reducing SSB consumption in rural Appalachian adults is currently being evaluated in a randomized controlled trial that includes a 2-group design (*i*SIPsmarter vs static Participant Education website) with 4 assessment points (Clinical Trial Registry: NCT05030753) [36]. When efficacy and other summative data are available, they will provide additional insights to inform the potential value of applying an HCD process to build *i*SIPsmarter as well as identify future areas of study.

**Six recommendations for the development or adaptation of digital behavioral health interventions.**

1. *Assemble a multidisciplinary team of experts and end users*: similar to our *i*SIPsmarter experience, the value of multidisciplinary team science and participatory processes in the development of digital behavioral health interventions is largely supported by other studies [15,16,18,35]. Our multidisciplinary content development team brought together expertise in the areas of nutrition content, behavior change, and rural health and worked alongside experts in digital behavioral health interventions, software engineering, instructional design, and user-interface design. Likewise, involving advisory team members with lived experiences in the targeted Appalachia region and with previous involvement in the SIPsmarter intervention brought immense value to the adaptation process. By applying human-centered design (HCD) principles [16], the advisory team members critically responded to iterative prototype versions, which helped shape key intervention decisions.
2. *Support efficient communication and decision-making processes among teams*: anticipating diverse feedback among different stakeholders, coordinating efficient communication among subteams, and finding a compromise are imperative to efficiently advance the HCD process [16,18]. For example, in our study, we coordinated communication and cooperation among 3 different subteams (ie, content, technology, and advisory teams) working to develop and advance *i*SIPsmarter. In some instances, the advisory team requirements were different and contradictory to one another and to the requirements of the content development team.
3. *Define areas of adaptation at the onset of the process*: Similar to the adaptation of any behavioral intervention, the adaptation of digital behavioral health interventions can be driven by several distinct purposes. For example, the Adaptome [70] describes 5 potential sources of intervention adaptations: core components, culture, mode of delivery, target audience, and service settings. We adapted *i*SIPsmarter from the evidence-based SIP smart ER trial with the clear goals of preserving the core components, cultural aspects, and the intended rural Appalachia target audience. This allowed us to concentrate on the mode of delivery and add key content around weight management. For example, we were able to focus end user feedback on potential digital divide concerns in Appalachia [21-26]. This feedback guided us to build *i*SIPsmarter with flexible features intended to enhance the likelihood of engaging in web-based cores, SMS text messages, and in-home weighing using cellular-enabled scales.
4. *Apply the available models and frameworks to guide digital intervention development processes*: Despite the promise and increased availability of digital behavioral health interventions, rapid disengagement and small effect sizes remain problematic [3]. Although evidence-based behavioral content is essential, it is only one of a multitude of factors that must be considered when developing or adapting digital behavioral health interventions. By applying the available models and frameworks to guide digital intervention development processes, researchers can identify and operationalize comprehensive components that affect the engagement and impact of digital behavioral health interventions [4-9].
5. *Clearly define instructional design goals to guide the HCD process*: To improve the likelihood of digital health applications meeting the needs and requirements of the users, instructional design objectives (eg, learning, affective, cognition, or psychomotor objectives) should be applied in the HCD process [15,16]. For example, in the *i*SIPsmarter interviews, we were interested in evaluating the participants’ knowledge, problem-solving skills, attitudes, and values associated with completing certain tasks (eg, navigating the dashboard, completing an action plan, and engaging with an interaction). By having clear instructional design objectives, we were able to better understand and modify *i*SIPsmarter features to meet the needs of the users and support the achievement of improved sugar-sweetened beverage consumption behaviors.
6. *Allocate appropriate resources and time to successfully execute HCD processes*: The time and resources required to develop or adapt digital health interventions can vary widely; however, they are often underestimated. For example, our iterative 3-phased HCD process was nearly an 18-month process. Researchers and developers should carefully consider and anticipate investment in robust HCD processes, as these processes are likely critical to the long-term uptake, engagement, and impacts of most digital health interventions.

### Limitations

Overall, 3 key limitations of this study should be considered. First, generalizability may be limited by the relatively small sample of advisory team members, the targeted rural Appalachia region, and SSB-specific content. Second, qualitative interviews can result in socially desirable responses. However, efforts were made by our trained interviewers to minimize this potential limitation by probing and clarifying the participant responses. This approach resulted in the participants providing thoughtful and constructive critiques of *i*SIPsmarter. Third, although we applied the *Model for Internet Interventions*, we limited the focus and scope of our manuscript to highlight the 8 main areas that comprise digital health applications [4]. Future efforts could focus more broadly on describing the other design-related components and elements that guided the *i*SIPsmarter adaptation process. Despite these potential limitations, we hope that the illustrated processes, scientific frameworks, and context-specific instructional design methodology will have wide applications in the development of digital behavioral health interventions across multiple geographic and behavioral contexts.

### Conclusions

Our process emphasizes the value of researchers and developers applying the existing models and frameworks as well as best practices in HCD and instructional design processes in digital intervention development processes. Together, the complementary skills of the CDT and advisory team members were invaluable in the *i*SIPsmarter adaptation process. The importance of the contextual and iterative prototype testing phases was largely reinforced by the overwhelming positive feedback received in the user testing phase. By illustrating *i*SIPsmarter content, we have highlighted the user-informed, high-quality products that resulted from our robust HCD process.

## Appendix

Multimedia Appendix 1 Goals and outcomes of the 13-series user-centered design process.

Multimedia Appendix 2 Example of a user’s action plan.

## Abbreviations

CDT: content development team
HCD: human-centered design
IVR: interactive voice response
RICE: Research Infrastructure Containing eHealth
SSB: sugar-sweetened beverage
