# Supplementary material for: Development of a Digital Behavioral Intervention to Reduce the Consumption of Sugar-Sweetened Beverages Among Rural Appalachian Adults: Multiphased, Human-Centered Design Approach
Source: JMIR Hum Factors. 2023 Feb 1;10:e41262. doi: 10.2196/41262 (PMC9932879; doi:10.2196/41262)
Supplement: Multimedia Appendix 1 [file humanfactors_v10i1e41262_app1.docx]

**Multimedia Appendix 1.** Goals and outcomes of a 13 series human-centered design process with Advisory Team participants.

| **Phase, Interview number, and Focus** | **Interview Objectives** | **Outcomes** |
| --- | --- | --- |
| Contextual phase  Interview 1: Technology | 1. Assess internet availability and use 2. Assess technology ownership and use 3. Understand technology preferences for an internet-based intervention 4. Identify barriers and benefits to receiving a technology-based sugar-sweetened beverage (SSB) intervention | - Participants were connected to the internet using multiple devices. They used the internet for different purposes.   Due to variations in internet access, technology accessibility must be considered in the design.  Email and short-message service (SMS) are both feasible options. Importantly, as participants engage with them differently, the modalities may serve different functions.  Noted barriers to an internet-based intervention included time, interest, and connectivity issues. |
| Prototype testing phase  Interview 2: Dashboard & Diary | 1. Assess comprehension of the online Dashboard 2. Assess comprehension of online Diaries 3. Evaluate understanding of SMS Diaries 4. Understand SMS preferences | - Most participants understood the Dashboard user interface, Core available email, and design elements (icons, colors, messaging). - The supportive resources for the SSB Diaries (drink card and paper diary) were well-designed and helpful due to having relevant examples and easy to understand information.   Participants valued the options for tracking and entering Diaries, understood how to edit SSB data on the Dashboard and liked receiving personalized feedback on their goal progress.  Having the option of an online Diary was viewed as important in case of poor cell service or missing the SMS.   - While there were mixed opinions on preferences for the timing of the SMS, participants overall reacted positively to receiving the daily messages due to convenience, ease of use, and clarity and supportive content in messages. |
| Contextual phase  Interview 3: Stepped Care and Weight-related questions | 1. Evaluate understanding of reminders to complete Cores 2. Collect feedback on stepped care timing and messages 3. Identify personal weight-related motivators and barriers 4. Understand perceptions around weight-related data and cellular-enabled scales | - Participants reacted positively to the stepped care process. They found the timing of contacts appropriate and the message content helpful to encourage completion of the Cores. - Participants valued an integrated weight monitoring tool. They thought tracking their weight could be motivating and provide accountability. - Noted barriers to weighing included privacy concerns, reluctance, and inadequate cellular service to transmit data. |
| Prototype testing phase  Interview 4: Personas | 1. Identify personas that are realistic, authentic, and relatable 2. Understand characteristics or qualities that make personas unrealistic or misrepresented 3. Identify SSB and weight trends that are realistic and relatable | - Participants found the personas to contain impactful stories and characters. - The impact of the personas was driven by them being realistic; incorporating relatable, representative, and diverse personas; highlighting different SSB patterns; and illustrating varied barriers and strategies to decreasing SSB. |

| Prototype testing phrase  Interview 5:  SSB Action Plan & Personas | 1. Identify areas of confusion when completing a SSB Action Plan 2. Evaluate understanding of SSB Action Plan steps 3. Assess comprehension of persona examples 4. Analyze ability to navigate buttons, dropdowns and multiple selection boxes | - Participants navigated the SSB Action Plan interface prototypes without difficulty and found steps clear. - The Action Plan personalized feedback and messaging were viewed as helpful to set SSB and weight goals. - Participants felt the action planning process allowed them to use their current SSB habits to make a pragmatic plan. - Persona examples helped illustrate the action planning process and provided helpful visual instructions on how to set goals. |
| --- | --- | --- |
| Prototype testing phrase  Interview 6:  SSB and Weight Action Plan & Personas | 1. Evaluate understanding and areas of confusing when completing steps of SSB and weight Action Plan 2. Assess comprehension of persona examples 3. Analyze ability to navigate buttons, dropdowns and multiple selection boxes 4. Assess comprehension of weekly goal and program goal setting | - Participants appreciated the insight offered by the interaction that allowed them to understand their potential weight loss over time by comparing their current SSB intake with their planned SSB reduction. - Participants liked the Action Plan modifications: more iconography, graphics, visuals and color coding. - The personas clearly illustrated key action planning steps and strategies to decrease SSB. |
| Prototype testing phrase  Interview 7: Weight Barriers & Strategies | 1. Evaluate clarity of weight barriers and strategies 2. Identify personal weight barriers and strategies | - Most weight-related barriers reflected barriers participants would choose or found relatable. - Participants felt the strategies to overcome barriers were clear and relatable. |
| Prototype testing phrase  Interview 8: Dashboard & Diary | 1. Assess comprehension of Dashboard and weekly tracking summaries 2. Evaluate understanding of online Diary 3. Determine usability and understanding of Dashboard icons | - Participants understood how to navigate and enter SSB and weight data on the Dashboard and Diary user interface. - Participants wanted to be able to visually see the status of their daily SSB and weight tracking on the Dashboard. |
| End user testing phrase  Interview 9: Getting Ready Core 1 | 1. Understand experience creating an account and accessing Core 2. Evaluate experience navigating Core content and interactions 3. Assess understanding of next steps following Core completion 4. Determine understanding of where to retrieve Core content and printable files 5. Understand experience tracking SSB and weight | - Participants found the Getting Ready Core enjoyable, easy to navigate, and appropriate in terms of language, use of images, and length. - Participants enjoyed interactive features, including interactions and vignettes. - Participants liked the personalization of setting their “own story” and reflecting on their typical SSB pattern.   The majority of participants liked the morning SMS because it was easy to reply and get an immediate verification message in return.   - Participants received the scale in the expected amount of time and were able to set it up and use it with few issues. - Participants identified bugs around scale usage and minor typos in the Core. |

| End user testing phase  Interview 10: Making a Plan Core 2 | 1. Identify barriers and strategies to tracking SSB and weight 2. Evaluate use of SMS to track SSB 3. Evaluate experience navigating Core content and interactions 4. Evaluate experience completing the SSB and weight Action Plan 5. Identify barriers and strategies to selecting weekly and program goals | - Participants tracked their SSB and weights using modalities that reflect personal preference for tracking and internet accessibility. - Participants had generally positive reactions to the Making a Plan Core in terms of length and inclusion of interactive and personalized features. |
| --- | --- | --- |
| Contextual phase  Interview 11: Enrollment procedures, call 1 | 1. Evaluate timing of first enrollment call 2. Determine best practices and procedures for first enrollment call 3. Identify areas for researcher improvement when conducting dietary recalls 4. Assess participant understanding of next steps in enrollment | - The majority of participants easily completed the interest screener in under 10 minutes. - Participants understood the iSIPsmarter consent form and found it helpful to be able to review it before the call. |
| Contextual phase  Interview 12: Enrollment procedures, call 2 | 1. Evaluate timing of second enrollment call 2. Determine best practices and procedures for second enrollment call 3. Identify areas for researcher improvement when conducting dietary recalls 4. Assess participant understanding of next steps in enrollment | - During the dietary recalls, participants found it helpful for the interviewer to provide context around the day recalled (e.g. yesterday, which was Tuesday). - Participants did not have any issues completing the baseline survey and completed it in less than 28 minutes. |
| End user testing phase  Interview 13: Remaining Cores and Wrap up | 1. Evaluate experience completing the iSIPsmarter Cores 2. Assess experience working towards personal goals 3. Determine ability to follow program recommendations 4. Identify strategies used to overcome barriers and meet personal goals 5. Analyze experience tracking SSB and weights 6. Identify benefits to participating in iSIPsmarter | - Most participants completed all 6 Cores. - Overall, they reported enjoying the program, especially the interactivities. - Parts of the program that were identified as very important included: (1) content related to reading labels, traffic-light system for food/drink, and SSB marketing; (2) tracking SSBs/weight; (3) vignettes; (4) maintenance action planning; and (5) the convenience of it being online. - Participants found strategies to track SSBs that worked for their technology needs. - Participants who weighed regularly reported losing weight. - Participants identified minor bugs and glitches that were resolved before launching the iSIPsmarter intervention. |
