# Supplementary figures and images for: Development of a Digital Behavioral Intervention to Reduce the Consumption of Sugar-Sweetened Beverages Among Rural Appalachian Adults: Multiphased, Human-Centered Design Approach
Source: JMIR Hum Factors. 2023 Feb 1;10:e41262. doi: 10.2196/41262 (PMC9932879; doi:10.2196/41262)

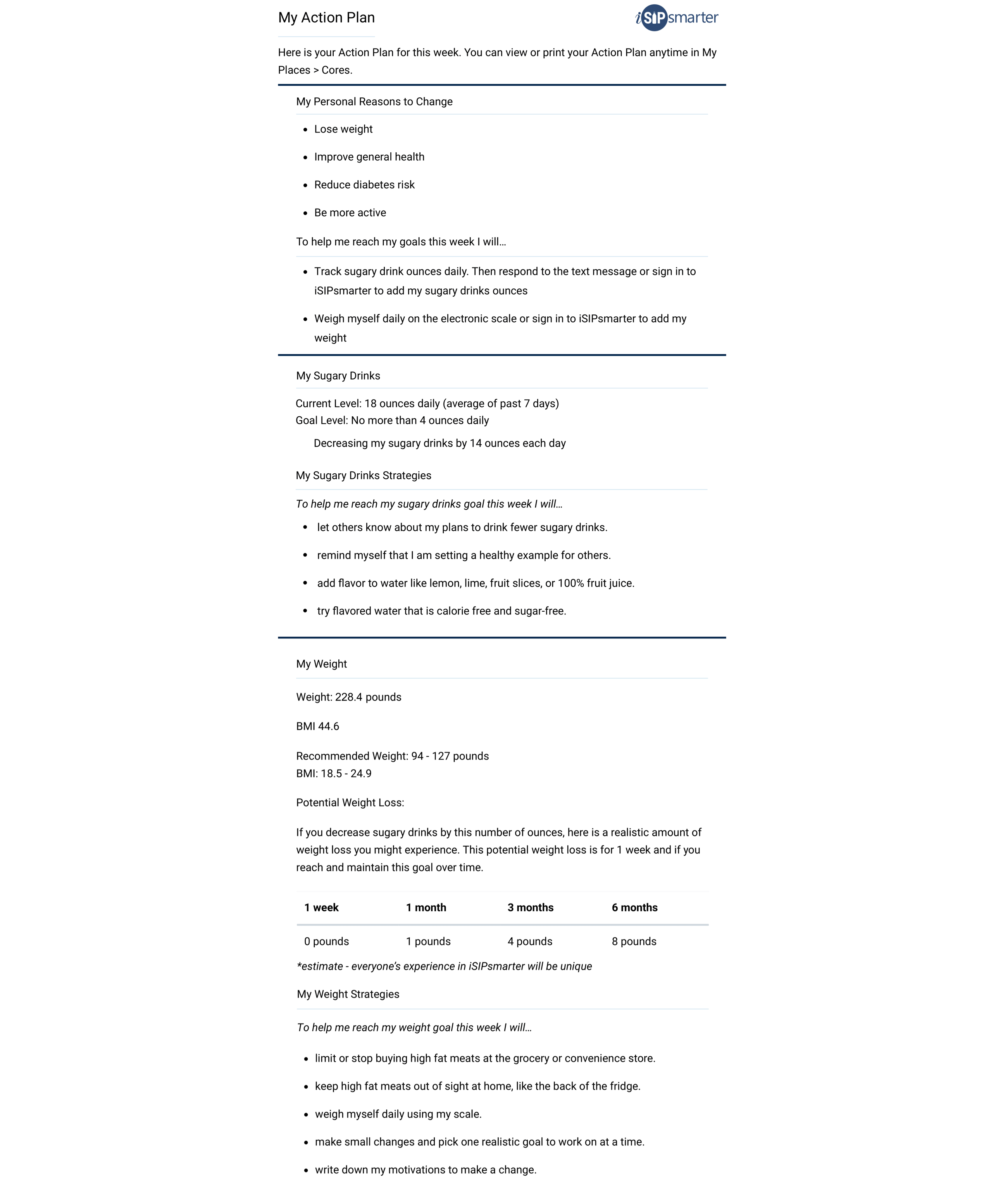

Supplement: Multimedia Appendix 2 [file humanfactors_v10i1e41262_app2.png]
